# Cardiovascular Impacts on COVID-19 Infected Patients

**DOI:** 10.3389/fcvm.2021.670659

**Published:** 2021-05-13

**Authors:** Somasundaram Raghavan, R. Gayathri, Sudhakar Kancharla, Prachetha Kolli, J. Ranjitha, Vijayalakshmi Shankar

**Affiliations:** ^1^Department of Pharmaceutical Sciences, University of Tennessee Health Science Center, Memphis, TN, United States; ^2^CO_2_ Research and Green Technologies Centre, VIT University, Vellore, India; ^3^Devansh Lab Werks, Homewood, AL, United States; ^4^Microgen Health Inc, Chantilly, VA, United States

**Keywords:** SARS-CoV-2, severe acute respiratory syndrome corona virus 2, COVID-19, ACE2, cardiovascular diseases, cardiovascular risk factors, cardiovascular system, cardiovascular abnormalities

## Abstract

The SARS-CoV-2 virus has taken more than 2 million lives on a global scale. Over 10 million people were confirmed with COVID-19 infection. The well-known spot of primary infection includes the lungs and the respiratory system. Recently it has been reported that the cardiovascular system and coagulation mechanisms were the second major targets of biological system affected due to the viral replication. The replication mechanism of SARS-CoV-2 involves the angiotensin-converting enzyme 2- (ACE2) surface receptors of endothelial cells belonging to various organs which act as the binding site for the viral spike (S) protein of SARS-CoV-2. The COVID-19 virus has been recently listed as a primary risk factor for the following cardiovascular conditions such as pericarditis, myocarditis, arrhythmias, myocardial injury, cardiac arrest, heart failure and coagulation abnormalities in the patients confirmed with COVID-19 viral infection. Direct and indirect type of tissue damage were the two major categories detected with cardiovascular abnormalities. Direct myocardial cell injury and indirect damage to the myocardial cell due to inflammation were clinically reported. Few drugs were clinically administered to regulate the vital biological mechanism along with symptomatic treatment and supportive therapy.

## Introduction

COVID-19 virus has created a global pandemic situation that had never seen by the world when compared to the other major infectious outbreaks. 2,244,713 patients were died due to COVID 19 based on the World health organization (WHO) report dated on 03.02.2021 (1). It has been clinically reported that the well-known primary target area of this virus is the lungs but several other tissues, organs, and organs system were also recorded to be severely injured by viral replication. Based on the clinical reports this virus mainly invades the respiratory system, the second major area of infection were recently recorded to be the cardiovascular system and blood coagulation mechanism. Other target areas include the gastrointestinal tract, kidney, and organs representing endothelial cell with ACE2 surface marker (2). The severity of these viral infections involves various factors including age groups, gender specificity, comorbid such as diabetes, hypertension, cardiovascular disease, suppressed immune system etc. (3). This review give emphasis on cardiovascular abnormalities caused by SARS-CoV-2 virus and its treatment possibilities.

## Cardiovascular Conditions Manifested With SARS-CoV-2 Infection

SARS-CoV-2 acts as a risk factor for developing the following cardiac abnormalities clinically reported until 2020 which includes Myocarditis, pericarditis, myocardial injury [MI], arrhythmias, and abnormal coagulation mechanism and these conditions were manifested asymptomatically in few cases (4). The death rate due to cardiac abnormalities was reported to be high in patients especially with myocardial injury indicated with an escalated level of troponin exhibiting a short life span.

## Factors Influencing Cardiac Abnormalities

The primary factor of risk include age, (elderly individuals), high blood pressure, diabetic clinical condition, hyperglycaemia, people diagnosed with congestive cardiac failure (CCF), cancer, coronary heart disease, Atherosclerotic heart disease act as absolute lethal factors in patients confirmed with SARS-CoV-2 (4).

## Symptoms

### General Symptoms for COVID-19 Infection

The symptoms for SARS-CoV-2 includes common symptoms associated with common cold and flu, intermittent diarrhea, anosmia, and ageusia (5, 6). The manifestation of symptoms was noticed 5 days after exposure to the viral particles (7). Dyspnoea indicates the severity of infection with clinical reports of elevated levels of plasma troponin, C-Reactive-Protein (CRP), Procalcitonin (PCT) and lymphocytopenia, IL-6, D-dimer and ferritin as a resultant product of blood mediated immune response due to hyperactivation of immune cells resulting inflammatory cytokine storm (8, 9).

### Cardiac Abnormalities Due to SARS-CoV-2 Infection

Chest pain representing the following heart conditions including ischemia, MI detected by the elevated levels of troponin, (CK) creatine kinase, natriuretic peptide (NT-Pro-BNP) were significantly noticed with few primary coronary occlusions. Both direct and indirect mechanisms of tissue damage were documented as a major cause for cardiomyopathy in the dead patient (10–12). Mortality caused by ventricular function and acute heart failure. Cardiomyopathy, asphyxia, ischemia, high metabolic demand, impairment of left ventricle, imbalanced electrolytes, proarrhythmic effect, consumptive coagulopathy, thrombogenesis in arteries as a consequence of hyperimmune response due to viral invasion were extremely recorded. Arrhythmia and cardiac arrest rank top position in cardiovascular abnormalities due to SARS-CoV-2 infection (13). The number of patients died due to cardiac arrest as a consequence of arrhythmia, and in the individual who was severely ill with COVID-19 were widely documented (14, 15).

## Various Mechanism Involved in Tissue Damage

Based on the mode of cellular damages caused to various tissues and organs, by viral replication mechanism, it has been categorized into direct and indirect modes resulting in cardiac and pulmonary damages (16).

### Direct Mechanism

COVID-19 virus can directly invade the cardiac and pulmonary tissues by using extreme target specificity (17). The damages caused by the viral replication to the cardiac and pulmonary region were detected with direct viral contact to the damaged cells. The SARS-CoV-2 virus makes use of the ACE2 surface receptors in order to establish the cellular contact in the cardiovascular, pulmonary system (18, 19). Thus, the viral entry into the host cell is facilitated by binding of S-protein (glycoprotein) with ACE2 surface receptors of endothelial cells belonging to various organs, chiefly the vascular endothelial cells and cardiomyocytes were directly invaded by SARS-CoV-2 (20).

#### The Angiotensin-Converting Enzyme

The angiotensin-converting enzyme (ACE) are protein units distributed on the surface of endothelial cells which act as a primary target for viral invasion into the cells belonging to the cardiovascular system, gastrointestinal tract, renal system and respiratory system and type 2 pneumocytes (4). Injured host cells belonging to the upper gastrointestinal tract (GI), heart, lungs, and kidney were detected in a dead patient infected with the SARS-CoV-2 virus (21, 22). This indicates that these tissues were directly injured by the viral replication mechanism (23–25).

#### Binding Mechanism

**Figure 1:**
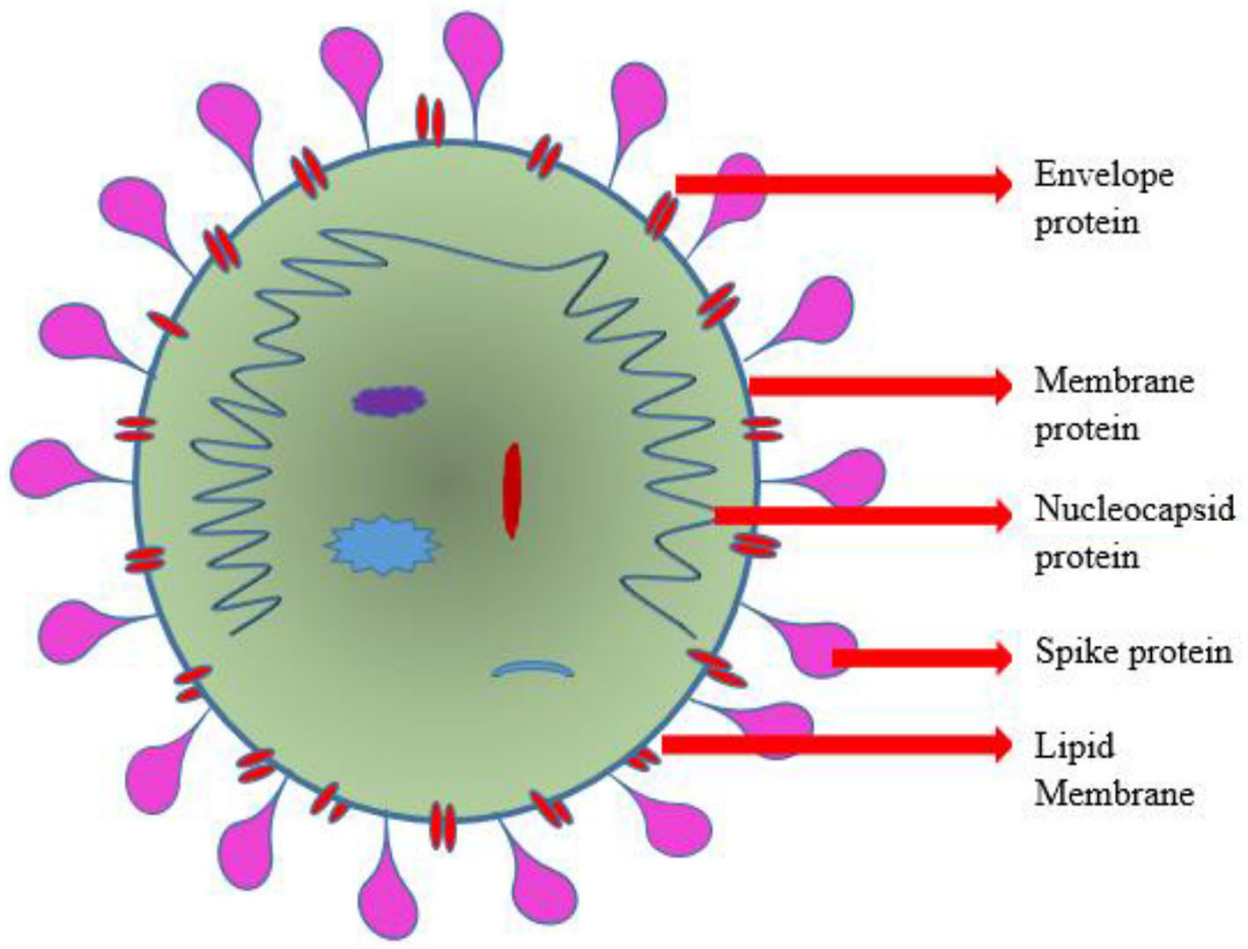
Schematic representation of the structure of coronavirus (SARS-CoV-2).

The viral S-protein recognizes the extracellular domain of this ACE2 type 1 integral protein-containing peptidase as a targeted binding site. The diagrammatic representation of SARS-CoV-2 was depicted in Figure 1. The membranous fusing of a host cell with viral spike protein is followed by the completion of binding (26, 27). Transmembrane Serine Protease 2 (TMPRSS2) plays a vital role in cleaving the binding site and priming of S-protein thereby facilitating the viral entry into the host cell (28–30). Phosphoinositide 5-kinase (PIKfyve), two-pore segment channel 2 (TPCN2), and cathepsin L were recently reported to be necessary for the passage of the virus into the host cell. Translation of polyprotein from viral RNA takes place, followed by assembly with genomic ribonucleotide to form virions (31–33). The transportation and release of virions from the damaged cell were carried out by the process of exocytosis. This intracellular viral replication suppresses and reduces the catalytic sensitivity of ACE2 surface molecules at its extracellular domain (34, 35).

### Indirect Mechanism

Systemic inflammation (SI) is the indirect mode of the viral mechanism causing damages to the cardiomyocytes. It has been reported that there is an elevated level of plasma troponin detected with a linear positive correlation with elevated CRP in blood. Procalcitonin (PCT) was also expressed along with CRP. This indicates that SI is a root cause for the damage of cardiomyocytes. Cytokine storm is also a part of an indirect mechanism leading to the damages of cardiomyocytes. Huang et al. (10) emphasized the biochemical reactions involved in cytokine storm detected in SARS-CoV-2 infected patients. It has been clinically observed that the balance between TH1 and TH2 was disturbed leading to cytokine storm ultimately resulting in the damage of cardiomyocytes. Once the post-infective cytokine signaling molecule was released it reduces the vascular flow in coronary arteries, lowers the O_2_ supply, and destabilizes the coronary plaque and formation of microvascular thrombosis. Persistent CVD (cardiovascular disease) becomes unpredictable and unstable due to an abnormal balance of high metabolic demand induced by a viral infection and decreased cardiac reserve. Acute Myocardial infraction (AMI-type 2) due to the uprise of metabolic demand by cardiomyocytes were documented in patients confirmed with SARS-CoV-2. There is an extreme chance for rupturing of coronary plaque and cardiac failure followed by SI in individuals with existing CAD (coronary artery disease). Abnormal coagulation has been recorded but the biochemical mechanism remains undiscovered. Arrhythmias were widely reported in COVID-19 infected individuals. Lymphopenia was highly associated with cytokine storm. Hypoxia, tachypnoea were few other clinical complications developed due to indirect viral mechanism. Multiple organ dysfunction Syndrome due to hyperimmune activity is a major cause of fatality (10).

## Cardiac Vascular Abnormalities By SARS-CoV-2 Infections

**Figure 2:**
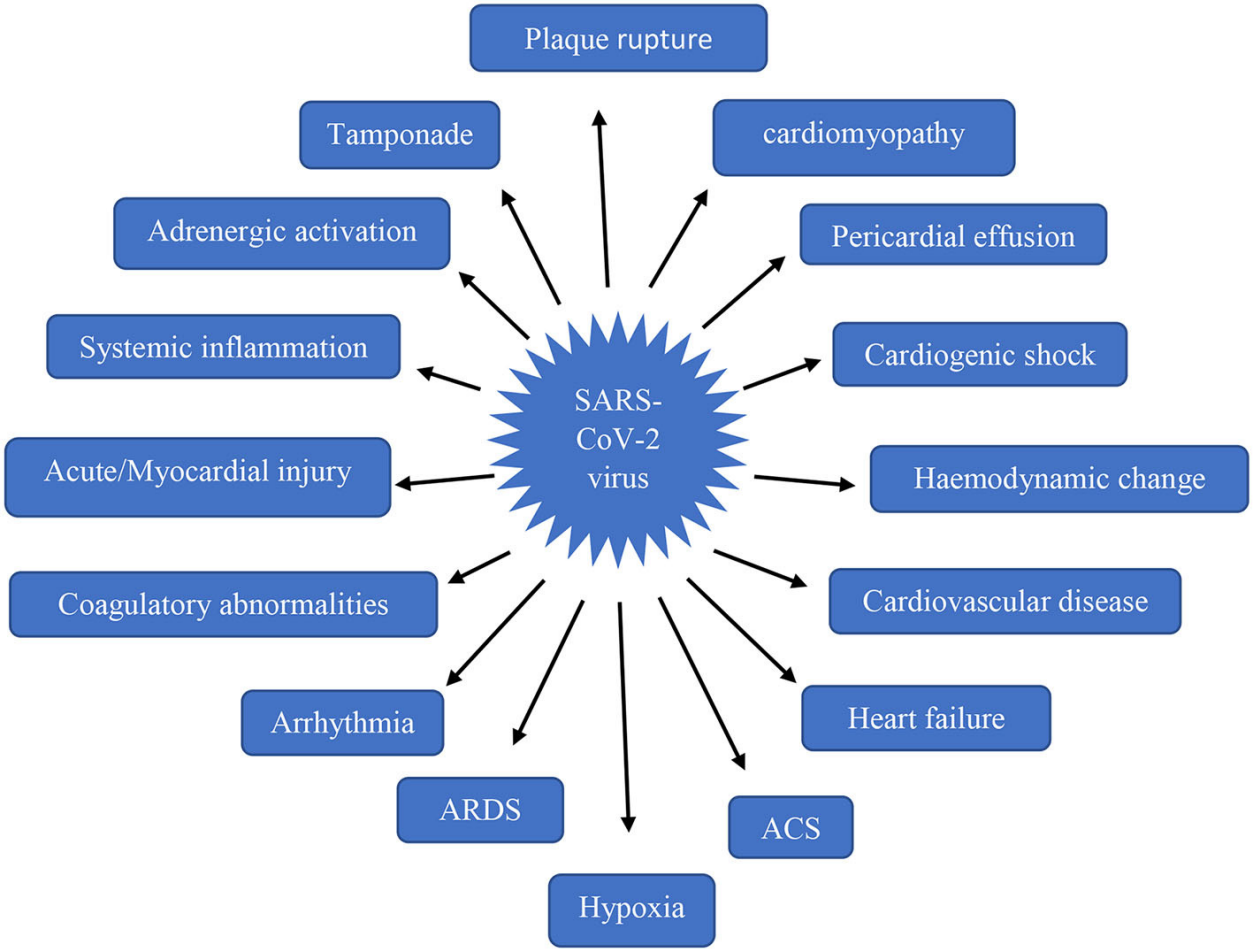
Cardiovascular conditions associated with SARS-CoV-2 virus.

Cardiovascular impairment arises in the stage only after the occurrence of respiratory distress. Most of the clinical conditions were reported due to a fall in O_2_ supply leading to tissue injury and cell death in the heart (36). The abnormalities by SARS-CoV-2 infection has been clearly shown in Figure 2. Direct viral entry into the heart occurs only after the pulmonary viral invasion, indirect damage mechanism also begins with triggering of a hyperimmune reaction during viral invasion into the lungs. Among the 50.1% inpatients, nearly 40.1% were reported with cardiovascular disorders (37, 38).

### Myocardial Injury

It has been clinically proven that SARS-CoV-2 itself causes acute MI and other cardiac disorders in patients without a history of any cardiovascular disorders (39, 40). Acute MI involves both direct and indirect viral mechanism resulting in the damage of cardiomyocytes (41). Among 52 patients with severe illness, 29% of the patients in ICU showed elevated levels of cardiac troponin 29 ng/L indicating acute MI (42, 43). The cardiac troponin was observed to be 29 ng/L in 1 out of 95 survivors and 34 dead patients showed the same levels out of 54 dead individuals. Among 8–12% of patients showed 99% of increased cardiac troponin with exacerbated impacts (12, 44). The mean difference of 25.6 ng/L was observed between patients with and without cardiac abnormalities after infection with SARS-CoV-2 (45, 46). Nearly 99% of the patient with cardiac abnormalities showed an intensification of previously diagnosed cardiac conditions due to the impact of the viral mechanism (26, 47, 48). Examination of tissues from the endocardium of an infected Italian inpatient was detected with acute MI, cardiac shock (CS), acute lymphocytic cardiomyopathy (49, 50). Numerous cases were diagnosed with cardiomyopathy associated with SARS-CoV-2 infection only with technical diagnostic images with the absence of histopathological analysis. Increased levels of biochemical markers such as creatine kinase, interleukin-6 (IL-6), transaminases, serum ferritin, lactate dehydrogenase, D-dimer along escalated prothrombin time noticed in dead patients implies that elevation of cytokine and substance mediating proinflammation reactions analogous to hypercytokinemia and systemic inflammation. Release of IL-6, Interferon gamma (IFN-_β_) occurs as a consequence of hypercytokinemia and Tumor Necrosis Factor-α (TNF-α), interleukin-1β (IL-1β), and IL-6 (51, 52). In Acute COVID-19 disease cardiovascular syndrome the degree of severity that the COVID-19 virus as the sole cause for acute MI, LV-dysfunction, increase in cardiac troponin remains uncertain. There is an unproven theory stating that micro-thrombogenesis as a consequence of abnormal coagulation mechanism would be serving as the main cause for MI but still, there is clinical evidence indicating the presence of pulmonary emboli. The pathophysiology cascade for injury and death of heart cells via direct damage is yet to be discovered (53).

#### Patterns of MI

In general, two types of patterns were recorded in tissue injury so far with COVID-19 infected patients.

##### Type 1

Manifestation of symptoms on 4th day of infection. Cardiac troponin levels were recorded to be >2.5–4.4 pg/mL and in patients who were alive and >8.7 pg/mL in dead patient. Subsequent rise in cardiac troponin was listed in the Table 1.

**Table 1:** Variations in the level of cardiac troponin in non-survived patients with MI (54).

**S. No**	**Biochemical marker**	**Levels of marker represented in pg/mL**	**No of days**
1.	Cardiac troponin	24.7	On day 7
2.	Cardiac troponin	55.7	On day 13
3.	Cardiac troponin	134.5	On day 19
4.	Cardiac troponin	290.6	On day 22

The duration between 15 and 20 days of infection was calculated to the statistically meantime for the death of the patients based on the death report of non-survived individuals. Other biomarkers levels suggested a cytokine storm as an additional mechanism and not MI as the sole cause of death.

##### Type 2

Infected individuals with a history of cardiovascular disorder showed the difference when compared to the pattern shown by patients with absence of CVD representing viral or stress cardiomyopathy was recorded (55). One patient showed chest pain with elevated ST-segment in the ECG report with the absence of a coronary occlusion. EKG reports indicated LV-impairment with 27% ejection fraction, along with troponin - 10.1 ng/mL, and NT-proBNP - >21,000 pg/mL, normalization of ejection fraction and biochemical markers were observed after 28 days when the patient is provided with steroids and Ig via IV line (56, 57). A Chinese man with the age of 63 without any CAD history was suffered from severe respiratory distress and fulminant cardiomyopathy showing LV enlargement of 6.1 cm, suppressed LV physiology with 32% ejection fraction, escalated troponin level more than 11 ng/mL and NT-proBNP around 22,000 pg/mL. ECMO along with Ig-IV, steroid, anti-viral and renal replacement therapy was administered to treat his cardiac shock based on the severity of his condition (58). Recovery of this patient was observed after 15 days with an improved ejection fraction of the left ventricle. The two patients were commonly treated with glucocorticoids but their effects remain uncertain and currently the use of glucocorticoids was terminated based on the guidelines issued by CDC and WHO. Palpitations and chest patient were reported among Chinese patients without common symptoms of SARS-CoV-2 (59).

### Cardiomyopathy or Myocarditis

Based on the data obtained from the meta-analysis of 1,527 patients included in 6 studies, around >8 % of inpatients were reported with acute cardiomyopathy. According to another meta-analysis with 4 studies with a sample size of 341 for an elevated level of cardiac troponin, analysis was reported with 36% out of 341 infected individuals. Continuous monitoring of cardiac troponin level from the onset of infection until recovery or death was highlighted by the investigator with the above mentioned statistical mean value (11, 60).

### Arrhythmia

From 138 hospitalized individuals nearly 44% was reported with arrhythmia (7, 38). One of the cardiovascular abnormalities to be highly recorded was acute myocardiopathy with 5.9% ventricular arrhythmia which includes ventricular tachycardia and ventricular fibrillation (61, 62). Arrhythmia may occur as a result of precipitation caused by imbalanced electrolytes (63, 64). Tachyarrhythmias was observed due to the fluctuation of Na with K levels associated with virus interaction with the renin-angiotensin-aldosterone system (65, 66). Based on the CDC report from the U.S.A around 18.5% of patients administered with mechanical ventilation developed atrial arrhythmias (67–69). The FDA (Food and Drug Administration) stated that chloroquine phosphate and hydroxychloroquine sulfate act as potential agents to induce arrhythmia (7, 70).

### Abnormal Coagulatory Mechanism

Venous thromboembolism, thrombogenesis in microvascular vessels were major clinical conditions due to abnormal coagulation mechanism clinically reported so far. The death rate of hospitalized individuals was observed to be high due to escalated D-dimer levels with >1 g/L. Comparative study of D-dimer levels between surviving and non-surviving hospitalized individuals showed 71.4% of non-survivors exhibited abnormal intravascular coagulatory mechanism indicated by Fibrin degradation product and d-dimer (71, 72). Thromboembolism can be strongly suspected in patients with severe illness as an outcome of hypoxia and hemodynamically unstable condition. Infected individuals immobilized for a longer period can give rise to (Disseminated intravascular coagulation) DIC and (Venous thromboembolism) VTE (2). In general tissue plasminogen activator (tPA) and plasminogen activator inhibitor-1 (PAI-1) activates the cascade for the lysis of fibrin. In the case of SARS-CoV-2 infected individuals, the following reactions will trigger the cascade for thrombogenesis which includes the mass discharge of cytokines or cytokine storm syndrome which triggers inflammatory reactions, activates the platelets, endothelial impairment, hypoxiation and disruption of haemostatics in infected individuals immobilized for a longer period (73).

### Other Cardiac Disorders

Arrythmia more than 44% of in patients in intensive care units was recorded with arrythmias along with 61% showed ARDS, 30.63% with cardiogenic shock with a sample size of 138 patients. From the critically ill 191 patients heart failure was noticed around 12% in severely ill individual and 52% dead individuals. Other complications include coagulation abnormalities with elevated levels of biochemical markers along with acute pulmonary embolism. Based on the report of CDC in china over 10% of people were died due to Cardiovascular abnormalities among 72,314. Around 44% of Patients recovered from SARS-CoV-2 infection with CVD history were recorded with worse outcomes of those clinical conditions (74, 75).

## Treatment

ACEi (Angiotensin-converting enzyme inhibitor) and ARBs (Angiotensin receptor blockers) are angiotensin receptors inhibitors and blockers used to inhibit the communicating between the host cell with the virus. In the earlier stage, it was uncertain but based on the recent data it was found to be useful for protecting acute pulmonary injury caused by internalization of SARS-CoV-2 with host cell-based on murine model test results (54). Anti-ACE2 molecules, TMPRSS2 inhibitor molecules and S1 protein subunits- inhibitors were experimentally proven to the potential agents for reducing viral replication inside the host cells (39). The coronary plaques were stabilized with drugs such as aspirin and statins (4). Symptomatic and supportive treatment requires administration of analgesia, antipyretic, O_2_ supply via a nasal cannula or Hudson mask based on the health condition of an infected person. Mechanical ventilation with low tidal volume is used to regulate hypoxia. Extracorporeal lung replacement procedure must be administered if invasive ventilation is insufficient (7–9). Administration of antiviral drugs such as lopinavir/ritonavir on 199 infected individuals was recorded with positive results showing significantly decreased viral load with improved symptoms. Remdesivir drug tested on cell lines also had a positive result with an inhibitory effect on the replicating mechanism of SARS-CoV-2 (76). The use of the above-mentioned antiviral drugs like hydroxychloroquine was discontinued for a short trial period due to the risk of developing hyperimmune reactions producing the clinical conditions - arrhythmia leading to death (77, 78). Individuals recovered from SARS-CoV-2 infection with MI were recommended not to undergo activities like exercise, sports and aerobics for 6–7 months to reach normal troponin levels confirmed with biochemical test with NMR diagnosis. Venoarterial extracorporeal membrane oxygenation (VA-ECMO) and extracorporeal membrane oxygenation (ECMO) were found to be useful for rescuing patients with refractory shock or ventricular arrhythmias as a result of acute COVID-19-cardiovascular syndrome. It has been clinically recorded that a patient was successfully saved with the use of VA-ECMO (46, 79).

## Conclusion

Recent reports of cardiovascular complications due to COVID-19 was clinically reported on a wide scale. Infection to SARS-CoV-2 has become a risk factor for cardiac disorders lately. Compared to adults, aged persons were prone to more complications and severity of cardiac conditions due to COVID-19 infection. Symptomatic and Supportive treatment was clinically administered for COVID-19 infected patients. Phase I and II clinical trials are currently in progress in many countries. The vaccine under clinical trials exhibits only short immunogenic effects which lead to administration of booster dose after 72–150 days to increase its efficacy up to 90%. To avoid infection preventive measures including usage of face mask, sanitiser, social distancing has to be followed until the discovery of a vaccine with prolonged immunogenic effects.

## Author Contributions

SR: conceptualization and investigation. RG: resources and validation. SK, PK, and JR: formal analysis. VS: conceptualization and supervision. All authors contributed to the article and approved the submitted version.

## Conflict of Interest

SK was employed by the company Devansh Lab Werks, Inc and PK was employed by the company Microgen Health Inc. The remaining authors declare that the research was conducted in the absence of any commercial or financial relationships that could be construed as a potential conflict of interest.
